# Could symptom burden predict subsequent healthcare use in patients with end stage kidney disease on hemodialysis care? A prospective, preliminary study

**DOI:** 10.1080/0886022X.2020.1744449

**Published:** 2020-03-25

**Authors:** Jing C. Zhang, Salam El-Majzoub, Madeline Li, Tibyan Ahmed, Joyce Wu, Mark L. Lipman, Ghizlane Moussaoui, Karl J. Looper, Marta Novak, Soham Rej, Istvan Mucsi

**Affiliations:** a Multi-Organ Transplant Program and Division of Nephrology, University Health Network, University of Toronto, Toronto, Canada; b Geri-PARTy Research Group, Department of Psychiatry, Jewish General Hospital, Lady-Davis Institute for Medical Research, McGill University, Montreal, Canada; c Princess Margaret Cancer Centre, University Health Network, Toronto, Canada; d Department of Nephrology, Jewish General Hospital, Montreal, Canada; e Centre for Mental Health, University Health Network, Toronto, Canada; f Department of Psychiatry, University of Toronto, Toronto, Canada

**Keywords:** Maintenance hemodialysis, Edmonton Symptom Assessment System-revised, end-stage kidney disease, healthcare use

## Abstract

**Context:**

Patients treated with maintenance hemodialysis experience significant symptom burden resulting in impaired quality of life. However, the association of patient reported symptom burden and the risk of healthcare use for patients with end stage kidney disease on hemodialysis has not been fully explored.

**Objectives:**

To investigate if higher symptom burden, assessed by the Edmonton Symptom Assessment System-revised (ESASr), is associated with increased healthcare use in patients with end stage kidney disease on hemodialysis.

**Methods:**

Prospective, single-center, study of adult patients on HD. Participants completed the ESASr questionnaire at enrollment. Baseline demographic, clinical information as well as healthcare use events during the 12-month following enrollment were extracted from medical records. The association between symptom burden and healthcare use was examined with a multivariable adjusted negative binomial model.

**Results:**

Mean (SD) age of the 80 participants was 71 (13) years, 56% diabetic, and 70% male. The median (IQR) dialysis vintage was 2 (1–4) years. In multivariable adjusted models, higher global [incident rate ratio (IRR) 1.02, 95% confidence interval (CI) 1.00–1.04, *p* = .025] and physical symptom burden score [IRR 1.03, CI 1.00–1.05, *p* = .034], but not emotional symptom burden score [IRR 1.05, CI 1.00–1.10, *p* = .052] predicted higher subsequent healthcare use.

**Conclusions:**

Our preliminary evidence suggests that higher symptom burden, assessed by ESASr may predict higher risk of healthcare use amongst patients with end stage kidney disease on hemodialysis. Future studies need to confirm the findings of this preliminary study and to assess the utility of ESASr for systematic symptom screening.

## Introduction

End stage kidney disease (ESKD) is a global health concern, affecting an estimated 2 million individuals world-wide [1,2]. In Canada, the prevalence of ESKD was 1300 per million population in 2016, which meant that ∼40 000 patients received treatment for ESKD in the country [3]. Many of the patients with ESKD require renal replacement therapy, that is either kidney transplant or dialysis; dialysis is most frequently utilized in Canada and in many other jurisdictions [3]. The average annual cost of in-center hemodialysis (HD) ranges from $60 000 to $100 000 (Canadian) [4,5], which poses a significant financial burden to healthcare funding. Patients on HD have a high rate healthcare use [hospitalization and emergency department (ED) visits] [6,7], that further increases the total healthcare cost associated with HD. Predictors of higher healthcare use in this patient population include age, socioeconomic status, marital status, and medical comorbidity [6,8–11]. Furthermore, depressive symptoms [12] and psychosocial distress [9,13] has also been associated with poorer survival and higher healthcare use.

Previous studies suggested that many patients with ESKD experience symptoms such as tiredness, pruritus, and constipation, pain, sleep disturbance, anorexia, anxiety, depression, restless legs, dyspnea, and nausea [14–17]. Self-reported symptom burden was associated with higher mortality, lower quality of life, and depression in patients on HD [15,18,19]. Symptom burden has also been associated with healthcare use in patients with cancer [20,21].

The Edmonton Symptom Assessment System-revised (ESASr) was developed and validated for patients with cancer [22–27] and was also validated among patients on HD [17,23,27]. ESASr has been used as a symptom screening tool in clinical cancer care and its use has been associated with a reduction in ED visits in patients with cancer [28,29]. A current study that explores the impact of systematic symptom screening and assessment of patient reported outcomes (PROMs) in HD care (EMPATHY trial) [30] employs ESAS-r (renal) for systematic symptom assessment.

Recent studies found an association between increased symptom burden and length of hospital stay [31] and mortality [32,33] in patients with cancer. However, the association between symptom burden and healthcare use in patients on HD has not been fully explored. In this study, we aim to analyze the association between symptom burden as assessed with the ESASr and the risk of healthcare use in patients on HD.

## Materials and methods

In this prospective, preliminary study we enrolled adults (>18 years of age) with ESKD receiving HD for more than 3 months at the Jewish General Hospital (JGH) in Montreal, Quebec, Canada. Patients were excluded if they had cognitive and sensory deficits that may have prevented them from completing the questionnaire, as determined by the healthcare team. We also excluded patients who were unable to understand English or French.

Enrollment began in July 2015. Abstraction of healthcare use data was started about 14 months after the first baseline assessment. Healthcare use data was extracted from participants’ health record for the 12 months period after baseline assessment. Clinical and socio-demographic data and healthcare use events (emergency room visits and hospital admissions) were extracted from the electronic medical records (EMR) of the JGH. Approval for this study was obtained from the Jewish General Hospital Research Ethics Board (‘Psychosocial Distress in Dialysis Study’, REB #15-046). Patients enrolled in the study provided written informed consent.

Symptom burden was measured at baseline using a paper/pencil questionnaire battery, that included ESASr. The questionnaires were presented in English or French as requested by the patient. ESASr measures symptom burden score on a Likert scale for 9-items (pain, tiredness, nausea, shortness of breath, lack of appetite, drowsiness, depression, anxiety and general wellbeing). Scores for individual symptoms range from 0 to 10 with higher score indicating worse symptoms [22–24,34,35]. Symptoms measured by ESASr were grouped into physical (pain, tiredness, nausea, shortness of breath, lack of appetite, drowsiness) and emotion (depression, anxiety) symptom burden scores by simple summation of individual symptom scores. We operationalized symptom burden into six variables, three variables were continuous symptom burden scores and three comprised of categorical global, physical, and emotional symptom burden.

Our primary exposure variables were the continuous ESASr symptom burden scores: global (9 items; theoretical range: 0–90), physical (6 items; theoretical range: 0–60), and emotional (2 items; theoretical range: 0–20) scores. We defined moderate/severe ‘global symptom burden’ if ESASr global symptom burden score was between 31 and 90 [36,37]. We could not find cut off scores for the ‘emotional’ and ‘physical’ sub-scales. In order to define patients with ‘moderate/severe’ symptom burden on these sub-scales, first we assessed the distribution of ‘global symptom burden’ scores of patients who were classified as having moderate/severe ‘global symptom burden’. The cut off score of 30 corresponded to the cut point for the highest quartile of the ‘global symptom score’ distribution. We then used the cut point that defines the highest quartiles for both the ‘emotional’ and the ‘physical’ sub-scale. Accordingly, we defined moderate/severe ‘physical symptom burden’ if ESASr physical symptom burden score was ≥19 and moderate/severe ‘emotional symptom burden’ if ESASr emotional symptom burden scores was ≥7. Moderate to severe individual symptoms were defined by the cut off >3 for all symptoms [17].

We defined healthcare use as the composite of ED visits and hospital admissions, as outcome variable. ED visits and hospital admissions during the one-year period following enrollment were extracted from electronic medical record. Outcome events and their corresponding dates, as well as censoring events (death, transplant, or loss to follow up) and their dates were extracted from EMR.

Potential covariables were selected based on their association with exposure or outcome, based on the literature and clinical experience. Sociodemographic (age, sex, marital status) and clinical (dialysis vintage, medical comorbidities, and hemoglobin level) variables were obtained from the EMR using a predesigned data abstraction form. Age, sex, marital status was cross-referenced with patient self-report. Dialysis vintage was categorized into three categories: ‘3–18 months’, ‘19–36 months’, and ‘>36 months’.

We report demographic, clinical characteristics and symptom prevalence using mean (standard deviation [SD]), median (interquartile range [IQR]), and proportions.

We assessed the associations between categorical demographic and clinical variables and categorical symptom burden using Fisher’s Exact test. We compared continuous variables between patients with none/mild vs moderate/severe global symptom burden using students’ *t*-test and the Mann Whitney *U* test, as appropriate. We described healthcare use as incident rates (IR; incidence per 100 person-year). Incidence rates were compared between groups by calculating incident rate ratios (IRR) and 95% confidence intervals (CI) using negative binomial regression to account for the skewed distribution of the data. Multivariable models were built to include potential confounding variables as determined from theoretical considerations and clinical experience: age, sex, marital status, dialysis vintage, and number of medical comorbidities. Two-tailed *p* values less than .05 were considered significant. All statistical analyses were performed on STATA v14.0 (StataCorp, College Station, TX).

## Results

**Figure 1.**
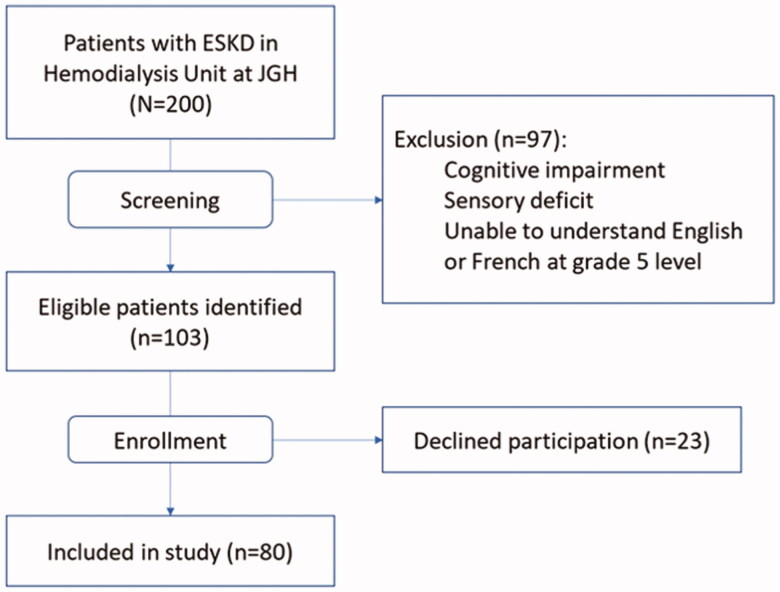
Flow diagram of participant selection (July 2015 to Aug 2016).

Among the 200 patients treated at the dialysis unit at the time of screening, 103 were potentially eligible for the study, and 80 (78%) provided informed consent (Figure 1). Mean (SD) age was 71 (13) years, the majority (70%) were male and married (68%). Thirty percent of participants requested the questionnaires in French. The median (IQR) number of comorbidities was 7 (4–8), and the median (IQR) dialysis vintage was 2 (1–4) years. More than half of participants had type 2 diabetes (56%) and hypertension (85%). Mean (SD) albumin level was at 39 (3) g/L and mean (SD) hemoglobin was 104 (10) g/L. Demographic characteristics of patients with none/mild vs moderate/severe global symptom burden did not differ significantly (Table 1).

**Table 1.** Baseline characteristics of the study sample.

		Total patient sample *n* = 80	None/mild global symptom burden *n* = 61 (76%)	Moderate/severe global symptom burden *n* = 19 (24%)	*p* Value
Mean (SD) Age (year)		71 (13)	72 (12)	71(15)	.782
Sex (male) *n* (%)		56 (70)	44 (72)	12 (63)	.568
Marital status (married) *n* (%)		54 (68)	40 (66)	14 (74)	.585
Dialysis vintage	3–18 months *n* (%)	29 (36)	21 (34)	8 (42)	.405
	18–36 months *n* (%)	22 (28)	19 (32)	3 (16)	
	36+ months *n* (%)	29 (36)	21 (34)	8 (42)	
Median (IQR) number of medical comorbidities		7 (5–8)	7 (5–8)	8 (4–10)	.62
Diabetes mellitus *n* (%)		45(56)	34 (56)	11 (58)	1
Hypertension *n* (%)		68 (85)	51 (84)	17 (89)	.721
Mean (SD) serum albumin (g/L)		39 (3)	39 (3)	38 (3)	.051
Mean (SD) hemoglobin (g/L)		104 (10)	104 (11)	104 (9)	.735
Edmonton symptom distress					
Total *n* (%) Moderate/severe global symptom burden (global symptom burden score >30)		19 (24)			
Median (IQR) global symptom burden score		16 (5–29)	10 (5–19)	43 (40–45)	<.001
Moderate/severe physical symptom burden *n* (%) (physical symptom burden score ≥19)		21 (26)	4 (5)	17 (21)	<.001
Median (IQR) physical symptom burden score		11 (5–20)	8 (4–13)	26 (21–30)	<.001
Moderate/severe emotional symptom burden *n* (%) (emotional symptom burden score ≥7)		22 (28)	6 (8)	16 (20)	<.001
Median (IQR) emotional symptom burden score		0 (0–7)	0 (0–1)	13 (8–16)	<.001
Median (IQR) prevalence of moderate/severe individual symptoms		2 (0–4)	1 (0–3)	6 (6–7)	<.001

SD: standard deviation; IQR: interquartile range; ESASr: Edmonton Symptom Assessment System-revised.

**Figure 2.**
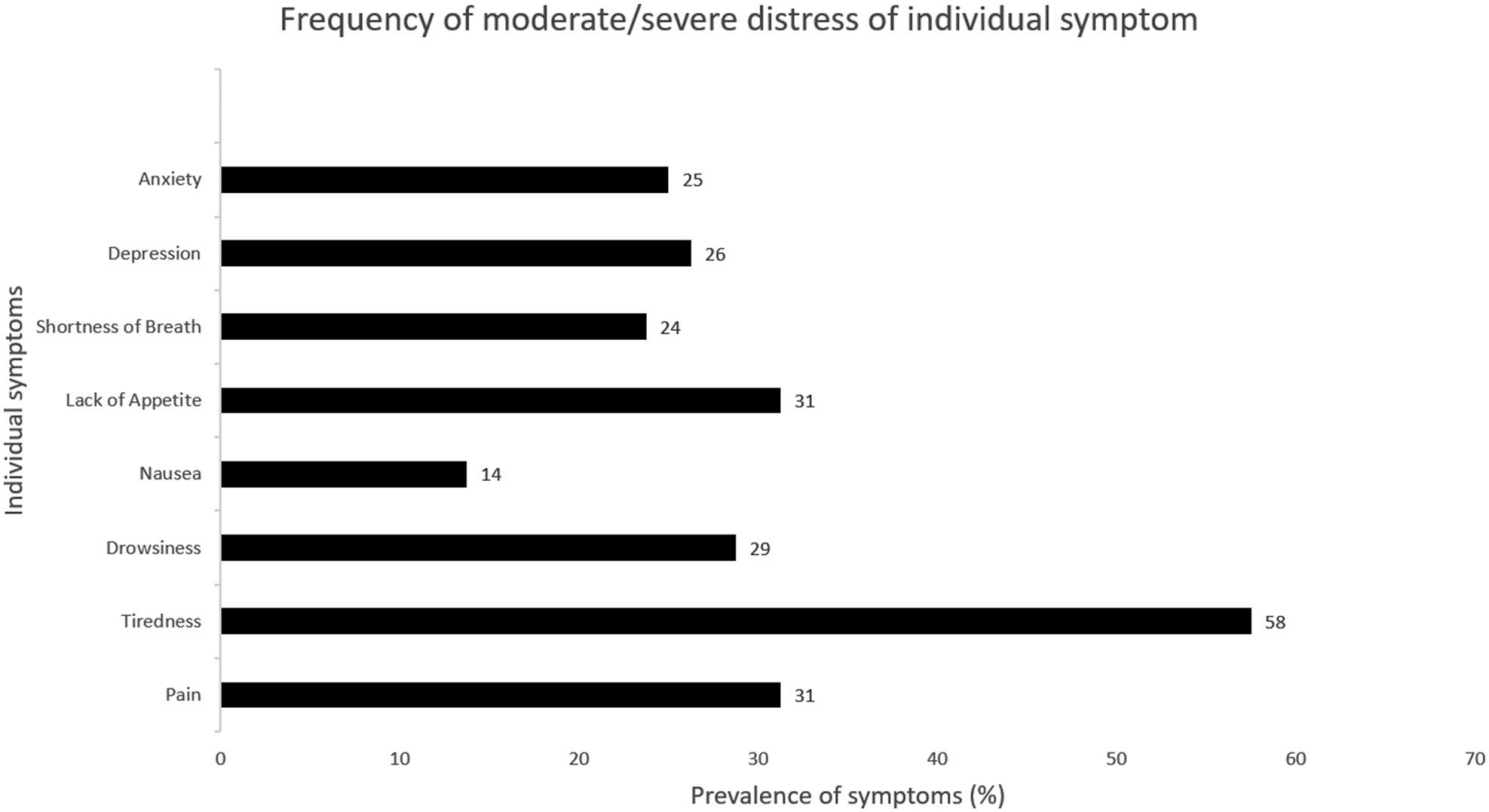
Prevalence of nine moderate/severe distress of individual symptoms in patients with ESKD.

The median (IQR) global symptom score was 16 (5–29), the median (IQR) physical score 11 (5–20) and the emotional score 0 (0–7). About one in four patients had significant (> =30) global symptom burden. Moderate/severe tiredness was reported by 58% of patients and was the most frequently reported symptom in our sample. Pain (31%), lack of appetite (31%), drowsiness (29%), depression (26%), and anxiety (25%) were all reported by more than one in four patients (Figure 2). In our patient sample, incidence rate (CI) of healthcare use was 72 (54–95) per 100 person-year.

In univariable negative binomial regression, higher ESASr global symptom burden score was not significantly associated with higher risk of healthcare use (Incidence rate ratio [IRR] 1.02, CI 1.00–1.03, *p* = .088). In a similar model, higher ESASr physical symptom burden score was near significantly associated with higher risk of healthcare use (IRR 1.03, CI 1.00–1.06, *p* = .064; Table 2). Additionally, emotional symptom burden score was not associated with healthcare use (IRR 1.03, 95% CI 0.98–1.08, *p* = .230). However, both higher global symptom burden score and higher physical symptom burden score were associated with higher risk of healthcare use after multivariable adjustment for sociodemographic and clinical covariables: IRR = 1.02 (CI 1.00–1.04, *p* = .025), IRR = 1.03 (CI 1.00–1.06, *p* = .034). Higher emotional symptom burden score was not associated with healthcare use in the multivariable adjusted model: IRR = 1.05 (CI 1.00–1.10, *p* = .052).

**Table 2.** Association between symptom burden and risk of healthcare use.

Incidence rate modeling (with adjustment)	Risk of healthcare use during the 12-month follow up					
Variables	Risk of healthcare use					
	Model 1			Model 2		
Edmonton symptom burden score	Incident rate ratio (IRR)	95% confidence interval (CI)	*p* Value	Incident rate ratio (IRR)	95% confidence interval (CI)	*p* Value
Global symptom burden score	1.02	1.00–1.03	.088	1.02	1.00–1.04	.025
Physical symptom burden score	1.03	1.00–1.05	.064	1.03	1.00–1.05	.034
Emotional symptom burden score	1.03	0.98–1.08	.230	1.05	1.00–1.10	.052
Edmonton symptom burden						
Moderate/severe Global symptom burden	1.37	0.73–2.56	.329	1.50	0.84–2.67	.169
Moderate/severe Physical symptom burden	1.75	0.98–3.16	.061	1.78	1.05–3.02	.032
Moderate/severe Emotional symptom burden	1.96	1.10–3.48	.022	2.32	1.38–3.91	.001

IRR: incident rate ratio; CI: confidence interval.

Model 1: Univariable analysis.

Model 2: Multivariable analysis: Model 1 + age, sex, marital status, dialysis vintage, number of comorbidities, and hemoglobin level.

In univariable negative binomial regression categorized moderate/severe global (IRR 1.37, CI 0.73–2.56, *p* = .329), and physical symptom burden (IRR 1.75, CI 0.98–3.15, *p* = .061) were not associated with healthcare use. However, moderate/severe emotional burden was associated with higher healthcare use (IRR 1.96, CI 1.10–3.48, *p* = .022). After multivariable adjustment, moderate/severe global symptom burden was not associated with healthcare use (IRR 1.50, CI 0.84–2.67, *p* = .169). However, moderate/severe physical (IRR 1.78, CI 1.05–3.02, *p* = .032) and emotional symptom burden (IRR 2.32, CI 1.38–3.91, *p* = .001) were both associated with increased risk of healthcare use after multivariable adjustment.

## Discussion

We report here that higher ESASr global and physical symptom burden score was associated with higher risk of healthcare use among patients on hemodialysis. However, when we assessed associations between categorized symptom burden and healthcare use, only moderate/severe physical and emotional, but not global symptom burden, was significantly associated with higher risk of healthcare use.

In our sample, higher global symptom burden score was associated with higher risk of healthcare use. These findings are in line with the results of Nipp et al. [38] who reported that higher global symptom score was associated with an increased length of hospitalization and a higher risk of unplanned hospital readmission in patients with cancer. We did not see statistically significant association when global symptom burden was categorized, defined by a cutoff score of >30 [36,37]. However, the cut off we used has been identified in patients with advanced cancer [36,37], not in patients on HD. Furthermore, we could not find published studies that assessed cut off points of ESAS scores amongst patients with ESKD. Therefore, in our study we used the cut off for ‘global symptom burden’ using published cut off for patients in an oncology cohort [1,2]. To define cut points for the ‘emotional’ and ‘physical’ sub-scales, we used a distribution-based approach. This resulted in a somewhat arbitrary cut off point for both ‘physical’ and ‘emotional’ symptom burden. We suggest that future studies should define appropriate cut points for ESASr scores for patients with end stage kidney disease. It is possible, that for patients with ESKD a different cut off, than the one suggested for patients with malignancy, will be needed. From our exploratory analysis, it appears that the cut off of >30 may be too high for patients with ESKD. Patients with ESKD have more significant comorbidities and may be more frail and vulnerable, compared to patients with malignancy. This may mean that they are at higher risk of using healthcare services at a lower level of symptom burden. This is suggested by the fact that when we explored various cut off scores near the sum of the physical (≥19) and emotional (≥7) cut offs (i.e., cut off 26 or 27), the association between global symptom burden and health care use was near significant (data not shown). We did not engage in more formal exploration of a cut off since our sample size was too small. Accordingly, additional studies in patients with ESKD may be required to identify disease specific cut off for the ESAS global symptom score. It is also possible that the lack of statistical significance in our analysis was due to small sample size.

Higher physical symptom burden score was associated with higher risk of healthcare use, independent of several potential confounders. Categorized moderate/severe physical burden was also associated with higher risk of healthcare use. Similar findings have been reported for patients with advanced cancer [38] where physical symptoms were associated with longer hospital stay and higher rate of hospital admission. These findings indicate that the ‘physical’ symptoms assessed by the ESASr may be a good representation of the overall clinical condition and symptom burden of the patient.

The ESASr emotional symptom score was near significantly associated with healthcare use, although the *p* value just above the conventional .05. However, categorized emotional symptom burden showed associations with healthcare risk. This latter result is consistent with findings reported by Abdel-Kader [39] and El-Majzoub [9] who showed that higher psychosocial distress predicted worse quality of life and higher healthcare use in patients with ESKD, respectively. More severe depressive symptoms were also shown to predict higher mortality in kidney transplant recipients [12]. In studies amongst patients with various chronic medical conditions, depressive symptoms are reportedly associated with increased risk of mortality, longer hospitalization, and higher risk of healthcare use [38,40–42]. These results from the literature suggest that in addition to physical symptom burden, emotional distress is also a risk factor for worse clinical outcomes and increased healthcare use in chronically ill patients.

In this sample, the median number of moderate/severe symptoms (cut offs >3) per patient was 2. This is lower than the average number of moderate/severe symptoms reported by others [17] using similar cut off. The low reported median frequency of moderate/severe symptoms in our patient sample could be due to the use of a different ESAS tool compared to Davison et al., who used a modified ESASr tool that included itchiness. Further contrary to Davison et al., we did not include ‘general wellbeing’ in the symptom count as it does not correspond to a specific symptom. More than half (58%) of our participants reported moderate/severe tiredness which is comparable to reported results in patients with ESKD on hemodialysis [14,43]. The frequency of other symptoms was all somewhat lower than reported for patients with ESKD on maintenance hemodialysis [14,15]. This may reflect an improvement of hemodialysis management during the last 10 years [44] potentially contributing to lower symptom frequency. Alternatively, lower than expected symptom frequency may be due to selection or reporting bias.

The incidence rate of healthcare use in our sample was also less compared to those reported by Ronksley et al. in patients with CKD [45]. This difference may be potentially explained by the selected nature of our convenience sample at a single center, whereas Ronksley et al. performed a population-based analysis.

ESASr may provide a useful snapshot of the symptom burden experienced by patients, it is brief and does not impose much inconvenience to patients. Furthermore, ESASr may identify significant symptoms that require specific management approaches. Further work, however, is still required to identify appropriate cutoff scores for ESASr to identify potential ‘cases’ who would benefit from further assessment or symptom management interventions. There are also potential concerns with this tool, since it uses a unidimensional approach in assessing complex symptoms, such as chronic pain, fatigue, anxiety, and depression. This may lead to reduced sensitivity, measurement precision and responsiveness. This may be potentially addressed using a two-step screening approach, like the one employed in the Distress Assessment and Response Tool currently used for routine distress screening at the Princess Margaret Cancer Center in Toronto, Ontario, Canada [13]. In that model, ESASr is used for initial symptom screening with a low threshold to trigger subsequent screening for depression or anxiety using more specific but still brief questionnaires. Potentially, combining ESASr with computer adaptive testing administration of the Patient Reported Outcomes Measurement Information System (PROMIS) item banks for depression, anxiety, fatigue, sleep disturbances may increase specificity of the assessment in clinical practice [46–48].

Several limitations of this study need to be considered when interpreting our results. First this is a single-center convenience sample, limiting the generalizability of our data. Second, the small sample size limited the statistical power and the number of potential confounders in our multivariable analyses. Third, we did not have ESKD specific cutoffs to categorize symptom burden or identify moderate/severe individual symptoms. Finally, we did not have information about the reason for ‘healthcare use’ in our dataset.

## Conclusion

In this study, we found that higher physical and emotional symptom burden is associated with higher healthcare use in patients with ESKD on maintenance dialysis. These results support the potential relevance of routine symptom assessment in dialysis centers to guide systematic symptom management in dialysis centers, to provide psychosocial and clinical support for patients with ESKD who have high symptom burden. When interpreting our results, however, it is important to consider the substantial limitations of the study. Therefore, we strongly suggest using these results as hypothesis generating for future studies. Specifically, studies are required to define condition specific cutoffs for moderate/severe symptom burden and for individual symptoms. Additional studies will also be needed to determine the exact magnitude of the risk of healthcare use attributable to symptom burden. Finally, the potential effects of systematic screening guided symptom management in patients on maintenance dialysis need to be investigated in the future prospective studies before a more reliable conclusion can be made.
